# Ultraviolet exposure regulates skin metabolome based on the microbiome

**DOI:** 10.1038/s41598-023-34073-3

**Published:** 2023-05-03

**Authors:** Vijaykumar Patra, Natalie Bordag, Yohann Clement, Harald Köfeler, Jean-Francois Nicolas, Marc Vocanson, Sophie Ayciriex, Peter Wolf

**Affiliations:** 1 Department of Dermatology, Medical University of Graz, Graz, Austria; 2 Centre International de Recherche en Infectiologie, Institut National de la Santé et de la Recherche Médicale, U1111, Université Claude Bernard Lyon 1, Centre National de la Recherche Scientifique, UMR5308, Ecole Normale Supérieure de Lyon, Université de Lyon, Lyon, France; 3 Université de Lyon, Université Claude Bernard Lyon 1, Institut des Sciences Analytiques, CNRS UMR 5280, 5 rue de la Doua, 69100 Villeurbanne, France; 4 Core Facility for Mass Spectrometry, Medical University of Graz, Graz, Austria; 5 Allergy and Clinical Immunology Department, Lyon Sud University Hospital, Lyon, France; 6 BioTechMed Graz, Graz, Austria

**Keywords:** Biotechnology, Computational biology and bioinformatics, Microbiology, Environmental sciences, Biomarkers, Medical research

## Abstract

Skin metabolites (< 1500 Da) play a critical role in barrier function, hydration, immune response, microbial invasion, and allergen penetration. We aimed to understand the global metabolic profile changes of the skin in relation to the microbiome and UV exposure and exposed germ-free (devoid of microbiome), disinfected mice (partially devoid of skin microbiome) and control mice with intact microbiome to immunosuppressive doses of UVB radiation. Targeted and untargeted lipidome and metabolome profiling was performed with skin tissue by high-resolution mass spectrometry. UV differentially regulated various metabolites such as alanine, choline, glycine, glutamine, and histidine in germ-free mice compared to control mice. Membrane lipid species such as phosphatidylcholine, phosphatidylethanolamine, and sphingomyelin were also affected by UV in a microbiome-dependent manner. These results shed light on the dynamics and interactions between the skin metabolome, microbiome, and UV exposure and open new avenues for the development of metabolite- or lipid-based applications to maintain skin health.

## Introduction

Exposure to sunlight, especially the ultraviolet (UV) component, is an important environmental factor affecting human health. UV radiation can penetrate the skin to the dermis (up to 200 µm) and cause both local and systemic changes in molecular and cellular components^1^. This can be therapeutically exploited in inflammatory skin diseases in humans on the one hand but can be harmful on the other leading to skin cancer and aging^2^. UV is a potent immunosuppressant, and the underlying immunological mechanisms are widely understood by now, but the impact of skin metabolites and lipids remains elusive.

Metabolites are low molecular weight compounds found in the skin that play a critical role in maintaining the homeostasis^3^. Skin metabolites originate from sweat, sebum (composed of lipids), interstitial fluid, protein degradation that occurs at the stratum corneum, and intracellular metabolites that play a role in immune responses^4^. Microbes that colonize the skin have a large reservoir of active enzymes that metabolize molecules and further influence the immune response^5^. In our previous work, we showed that the presence or absence of a microbiome determines the effect of UV radiation on cutaneous immune response^6^. In the current study, we investigated the effect of UVB exposure on global skin metabolites and lipids depending on the presence or absence of the microbiome and performed metabolomics and lipidomics analysis on skin biopsies. To investigate if the effects of UV exposure on skin metabolites are dependent on local microbiome or distal gut microbiome, we either used germ free (GF) mice or locally disinfected the UV exposed skin of normal mice. We report a global change in the skin metabolomic and lipidomic profiles induced by UV exposure, which depends on the presence or absence of the skin microbiome.

## Results

**Figure 1:**
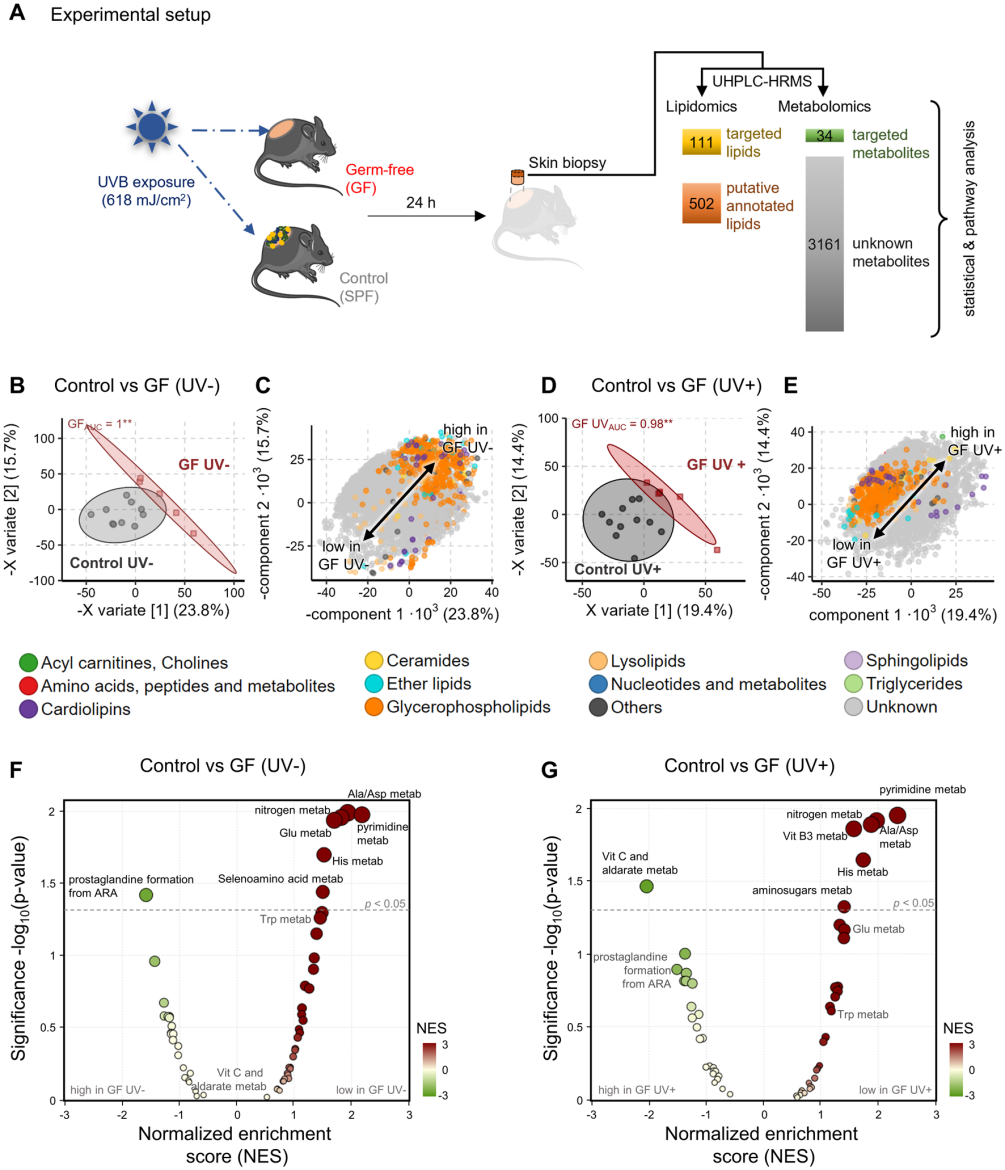
Cutaneous metabolome strongly depends on the presence of microbiome before and after UVB exposure. (**A**) Schematic overview of study design. Skin biopsy samples were subjected to metabolomics and lipidomics measurements from which data was extracted in a targeted and untargeted approach as described in methods. (**B**) PLS-DA scores plot investigating the difference of cutaneous metabolome in the absence of any microbiome (GF) before UVB exposure compared to control mice. Each point represents the metabolome of one mouse skin sample and nearness of points represents metabolic similarity. ROC analysis with X variate 1–3 found the metabolomes to differ significantly (p < 0.001) with an AUC of 1. The 95% confidence interval of each group is marked by their coloured ellipse. (**C**) Corresponding PLS-DA loadings plot to (**B**). Each point represents a metabolite’s contribution to the group separation observed in the scores plot (**B**) showing that unknown metabolites strongly differ after UVB exposure. (**D**) PLS-DA scores plot investigating the difference of cutaneous metabolome in the absence of any microbiome (GF) after UVB exposure compared to control mice. Points and ellipse as in Panel B. ROC analysis with X variate 1–3 found the metabolomes to differ significantly (p < 0.01) with an AUC of 0.98. (**E**) Corresponding PLS-DA loadings plot to (**D**) [points = metabolites as in (**B**)] showing that unknown metabolites strongly differ after UV exposure. (**F**) Functional analysis of unknown metabolites in the absence of any microbiome (GF) before UVB exposure compared to control mice. Only significantly impacted pathways are labelled (p < 0.05). (**G**) Functional analysis of unknown metabolites in the absence of any microbiome (GF) after UVB exposure compared to control mice. Significantly impacted pathways are labelled above the dotted line (p < 0.05). N = 6–10 mice per experimental group. Data is pooled from two independent experiments.

A single immunosuppressive UVB dose (618 mJ/cm^2^) to the skin induces a different transcriptomic signature, cellular infiltrate, and immune response in GF mice compared with control mice^6^. We therefore hypothesized that a similar dose of UVB might also alter the overall metabolic and lipidic profile of the skin, which may contribute to immune modulation in dependence of the microbiome. To test this, we used skin samples from GF mice (n = 6; samples pooled from two independent experiments), mice with depletion of local microbiome by disinfection (n = 5), and control mice (n = 10; samples pooled from two independent experiments), obtained 24 h after UVB exposure (Fig. 1A). The cutaneous metabolome was investigated with targeted and untargeted high resolution mass spectrometry. For statistical analysis a total of 111 targeted lipids and 34 targeted metabolites were extracted, whereas the untargeted data extraction yielded 502 putatively annotated lipids and 3161 unknown features (Fig. 1A).

The absence of microbiome (GF) strongly shifted the cutaneous metabolome compared to control mice with intact microbiome, both before (Fig. 1B,C) and after UVR exposure (Fig. 1D,E) which can be seen from the clear clustering and separation of groups in the supervised multivariate method PLS-DA. Similar results were seen in skin of disinfected mice compared to control mice (Fig. S1A,C). At baseline (unexposed) the metabolic difference is mainly driven by higher lipid levels such as cardiolipins (CL), phosphatidylcholines (PC), phosphatidylethanolamines (PE) or phosphatidylserine (PS) and lower unknown metabolites (Fig. 1C) whereas after UVB exposure it is driven mainly by increase in unknown metabolites (Fig. 1E), comparing GF mice vs. control mice. Independent of the microbiome, the UVB exposure brings the cutaneous metabolomes closer together, as the explained variability (sum of the first two X variates) decreased from 39.5% before (Fig. 1B) to 33.8% after UVB exposure (Fig. 1D). The metabolic shift was notably similar in disinfected skin where the explained variability decreased from 38% before (Fig. S1A) to 36.8% after UVB exposure (Fig. S1C). Analog to GF mice the metabolome of disinfected skin differed significantly from that of control skin before and after UVR exposure (Fig. S1A/C) in both cases mainly driven by unknown metabolites and few increases in glycerophospholipids (Fig. S1B/D). UVR exposure reduced the metabolic differences between non-disinfected skin and disinfected skin, best visible from the univariate LOGLME analysis (Fig. S1E).

Mummichog automated annotation and pathway analysis was used to generate more detailed insights, since most statistically significant metabolic differences were observed in unknown metabolites. Out of 3161 unknown features, 1380 were matched by mummichog for pathway analysis. The pathway analysis revealed that the absence of microbiome consistently diminishes the activity histidine, pyrimidine and alanine/aspartate metabolism among others compared to control mice before (Fig. 1F) and after UVB exposure (Fig. 1G and Supplementary Table S1).

**Figure 2:**
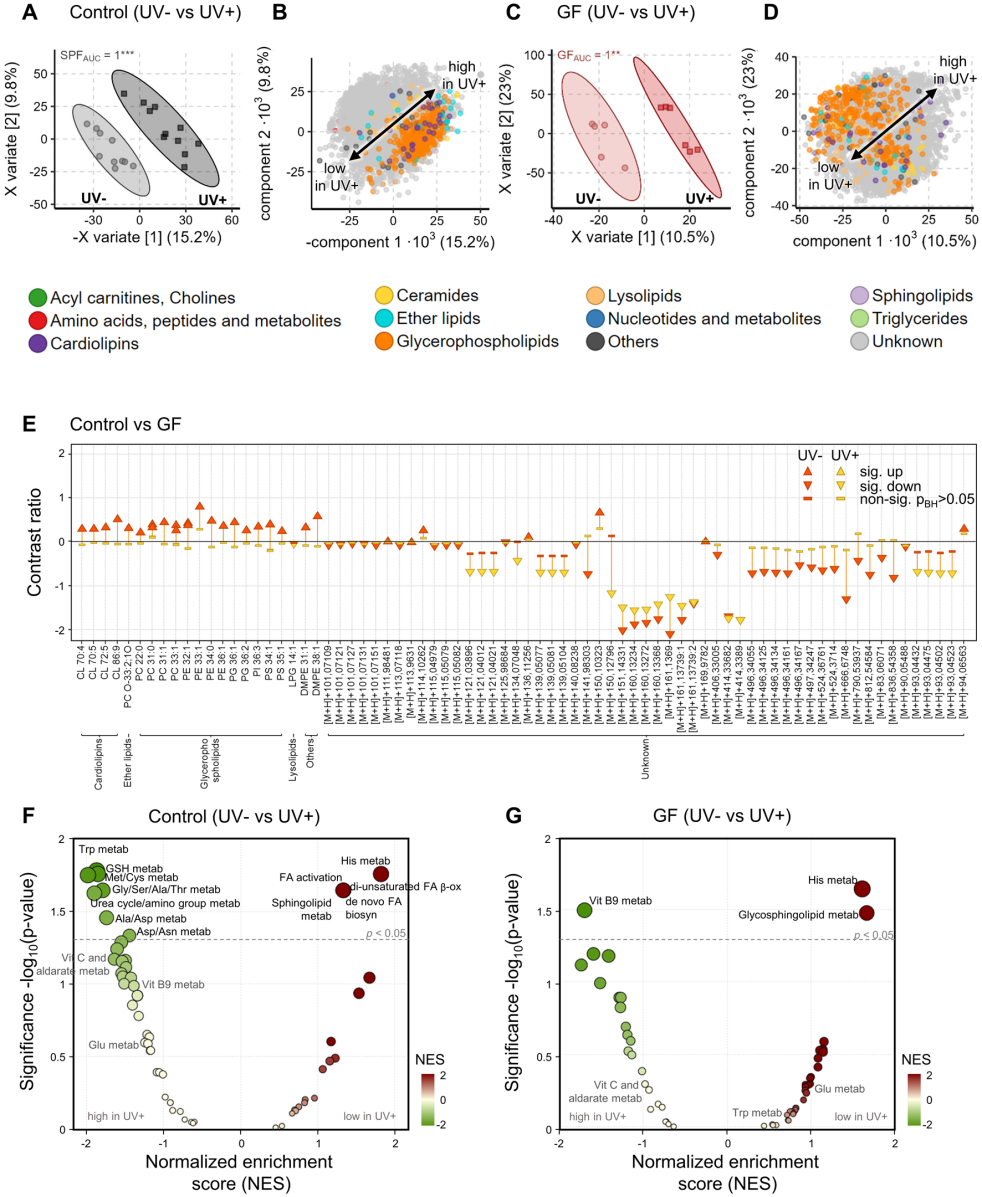
UVB exposure reduces intra-cutaneous metabolic differences induced by presence/absence of the microbiome. (**A**) PLS-DA scores plot investigating the difference of cutaneous metabolome induced by UVB exposure in control mice. Points and ellipse as in Fig. 1. ROC analysis with X variate 1–3 found the metabolomes to differ significantly (p < 0.01) with an AUC of 1. (**B**) Corresponding PLS-DA loadings plot to (**A**) (points = metabolites as in Fig. 1) showing that unknown metabolites strongly differ after UV exposure. (**C**) PLS-DA scores plot investigating the difference of cutaneous metabolome induced by UVB exposure in GF mice. Points and ellipse as in Fig. 1. ROC analysis with X variate 1–3 found the metabolomes to differ significantly (p < 0.01) with an AUC of 1. (**D**) Corresponding PLS-DA loadings plot to (**C**) (points = metabolites as in Fig. 1) showing that unknown metabolites strongly differ after UV exposure. (**E**) Dumbbell plot of all significant LOGLME metabolites (p < 0.05) in any of the two comparisons: control mice vs GF before UVB exposure (orange) or after UVB exposure (yellow). The plot shows the strength of metabolic changes along the y-axis, significance is encoded in shapes (p < 0.05) indicating significant increase (sig. up), decrease (sig. down) or non-significant (non-sig). Note how there are much fewer significant differences after UV exposure (yellow). (**F**) Functional analysis of unknown metabolite changes induced by UVB exposure in control mice. Significantly impacted pathways are labelled above the dotted line (p < 0.05). (**G**) Functional analysis of unknown metabolite changes induced by UVB exposure in the absence of any microbiome (GF). Only significantly impacted pathways are labelled (p < 0.05). N = 6–10 mice per experimental group. Data is pooled from two independent experiments.

UVB exposure itself induced a strong and significant shift in the overall cutaneous metabolome in both the control mice (Fig. 2A) and the GF mice (Fig. 2C) which was mostly driven by unknown features (Fig. 2B,D). In control mice with an intact microbiome a few selected lipids (e.g., PE O-38:4, PC O-34:0;3O) were increased after UV exposure (Fig. 2B). A detailed analysis with univariate LOGLME underlines how many single significant metabolic differences between control and GF mice become less pronounced or non-significant after UVB exposure (Fig. 2E). The mummichog based pathway analysis shows that in control mice UV exposure increased amino acid metabolism, e.g., Trp, Gly, Ser, Ala, Thr, Ala, Asp, Asn, as well as their amino group metabolism in the urea cycle, while histidine metabolism was decreased together with fatty acid (FA) and sphingolipid metabolism (Fig. 2F). In GF mice fewer pathways were significantly impacted, indicating that the lack of microbiome and microbial metabolism, ameliorates the reaction to UVB. Mainly vitamin B9 metabolism increased, a light sensitive vitamin required for DNA synthesis, while glycosphingolipid metabolism decreased and as in control mice also histidine metabolism decreased (Fig. 2G). We observed similar results in disinfected mice (Fig. S2C) indicating the role of local microbiome in regulating UV-induced metabolic pathways.

In disinfected skin of mice, like in control or GF mice, the UVR exposure significantly and strongly shifted the metabolome (Fig. S2A) which was mainly driven by unknown metabolites and increases in some glycerophospholipids (Fig. S2B). The metabolic shift was notably stronger in disinfected skin than in the skin of control and GF mice judged by the higher x variate percentages of the first two components with a sum of 25.0% control (Fig. 2A) vs. 33.5% GF (Fig. 2C) vs. 47.6% disinfected mice (Fig. S2A).

Pathway analysis further showed that skin disinfection lowered mainly amino acid (Arg, Pro, Asp, Asn) metabolism as well as lipidic mediators-related pathways such as ARA metabolism or putative anti-inflammatory metabolism from EPA. In contrast to the skin of GF mice, histidine metabolism was not significantly impacted in disinfected mice, although trending in the same direction towards being decreased (Fig. S1F and Supplementary Table S1). After UVR exposure the decrease in histidine metabolism became highly significant in disinfected mice (Fig. S1G), in a similar fashion as for GF mice (Fig. 1G). However, the other decreased pathways differed such as urea cycle amino group, Asp, Asn, Arg, Pro, β-Ala amino acid or vitamin B9 metabolism.

Summarized, the cutaneous metabolome strongly depends on the microbiome as seen in unexposed GF and control mice, while UVB exposure reduces microbiome driven metabolic differences.

## Discussion

It is well known that the microbiome plays a critical role in maintaining immune homeostasis and is involved in numerous processes in the skin^7^. To our knowledge, no studies have been performed to understand the complex relationship between UV exposure, skin microbiome, metabolome, and lipidome. Here, we report an altered profile of the global skin metabolome before and after UVB exposure, depending on the microbiome. UV exposure can affect lipids both locally and systemically. A single acute UV irradiation (2–3 MED) is sufficient to induce changes in the lipid profile in the skin, as previously reported^8^. Similarly, we observe changes in both lipid and metabolic profiles in the skin after acute UVB irradiation (2 MED); moreover, we show that these changes depend strongly on the presence or absence of the microbiome. Lipids and lipid metabolism are increasingly recognised as a crucial factor in immune modulation^9^. It is known that several species of the skin microbiome contain enzymes capable of utilising lipids and altering their concentrations on the skin, which could potentially contribute to immunomodulation^10^. Our results shed new light on this phenomenon and support this concept.

Metabolites play a critical role in immunomodulation^11^. Previous studies have focused exclusively on examining specific classes of metabolites^9^, whereas here we provide a much broader and holistic view of the UV-induced metabolic profile of the skin as a function of the microbiome. In the absence of the microbiome, we observe increased ascorbate (vitamin C) and aldarate metabolism in UV-exposed germ-free mice compared to control mice with an intact microbiome. Vitamin C is known to attenuate photoaging, stimulate collagen synthesis, provide antioxidant protection, and have great cosmetic potential^12^. In addition, the vitamin B9 or folic acid vitamin pathway was also higher in UV-exposed germ-free and disinfected mice. Folic acid is known to reduce oxidative stress, increase skin hydration, and improve barrier function^13^. On the other hand, we observed lower glycosphingolipid metabolism in UV-exposed germ-free mice. It is known that sphingolipid metabolites play a key role in the regulation and infiltration of immune cells during inflammation^14^. These altered metabolic pathways could contribute to the enhanced UV-induced immunosuppression in germ-free and disinfected mice. In the presence of the microbiome, we observe enhanced metabolic pathways such as alanine and aspartate, pyrimidine, nitrogen, glutamate, histidine, and selenoamino acid metabolism. Interestingly, all these metabolic pathways are associated with microbial metabolism^15^ and could explain the increase in specific metabolites associated with these pathways in unexposed control mice with intact microbiome compared with germ-free mice. In addition, UV exposure increased tryptophan metabolism in control mice. Although it is difficult to tell from our data whether UV radiation caused an increase in tryptophan metabolites produced by the microbiome or by skin cells. Nevertheless, tryptophan metabolites may exert a regulatory function in inflammation and contribute to UV-induced inflammation in the skin^16^. Other metabolic pathways such as glutathione, methionine and cysteine, glycine, serine, alanine and threonine, urea cycle/amino group, alanine, aspartate, and asparagine metabolism induced by UV exposure in control mice may be related to decreased immunosuppression, increased epidermal thickness and cellular infiltrate, and a proinflammatory environment.

### Limitations

We used a single, rather high UVB dose (causing systemic immunosuppression); thus, whether lower UVB doses could produce similar results remains to be determined. That said, previous reports of human skin with moderate to severe photodamage show a globally altered metabolic profile^17^. A single proinflammatory UV dose (3 MED) modulated skin lipids for up to 14 days post exposure^8^. In vitro experiments with cultured keratinocytes exposed to 20 mJ/cm^2^ of UVB radiation showed altered metabolic activity^18^. Taken together, these results suggest that the skin metabolic profile may be dependent on the dose and/or duration of UV exposure. We used a single technique (HPLC–MS) to assess metabolites and lipids and it thus would be desirable to further confirm these specific metabolites and lipids using other advanced techniques such as MS based imaging in the tissue sections^18,19^. The unexposed skin of GF and disinfected mice differed with regard to certain metabolites and metabolic pathways. We hypothesise that this difference may have been due to the type of disinfectants used, which could have microbiome-independent effects on the metabolome. Indeed, it has been reported that disinfectants or their by-products can alter cellular metabolites^20^. Moreover differences in microbial load can also influence the metabolic profile of the skin^21^.

## Conclusion

Our data provides a good basis for understanding the complex interactions between UV exposure, skin microbiome, metabolome, and lipidome. These results warrant further studies looking in more detail at UV- and/or microbiome-induced specific metabolites or lipids and their effects on the immune response. Identifying such metabolites could lead to development of novel strategies to interfere with specific metabolic pathways to maintain skin “health” after UV exposure.

## Methods

### Animals

Skin samples were available from animal experiments that were previously performed^6^. Protocols involving the use of germ-free animals were approved by the Regional Animal Research Ethical Board, Stockholm, Sweden (Stockholms norra djurförsöksetiska nämnd), following proceedings described in EU legislation (Council Directive 86/609/EEC). Animal husbandry was done in accordance with Karolinska Institutet guidelines and approved by the above-mentioned board (Ref: N190/15)^6^. Animal experiments adhered to 3R (replacement, reduction, and refinement) policy to ensure use of minimum numbers of animals to maximize data mining^6^. The reporting in the manuscript follows the recommendation in the ARRIVE guidelines.

### Skin disinfection model

Shaved dorsal skin of mice was disinfected, as previously described^6^, using freshly prepared 10% or 2% chlorhexidine and 70% isopropyl alcohol, 24 h and 1 h before UVB irradiation and the cages of animals were changed every day through the experimental protocol. Animal care and treatment protocols were approved by the Austrian Federal Ministry for Science and Research, through protocol number BMWFW-66.010/0137-WF/V/3b/2014. Animal experiments adhered to 3R (replacement, reduction, and refinement) policy to ensure use of minimum numbers of animals to maximize data mining.

### UV-B source and exposure

UV irradiation protocol was performed as previously described^6^. The backs of the mice were shaved with electric clippers 24 h before irradiation. UV radiation was administered using a Waldmann 236 light source (Waldmann Medizintechnik, Villingen-Schwenningen, Germany). The light source was equipped with two Waldmann UV6 fluorescent tubes (emission range 280–360 nm; peak 320 nm). The UVB device was positioned upside down on the cage. The mean UVB irradiance of the lamp was 1.9 mW/cm^2^, measured with a Waldmann UV photometer with a UV6 detector head matching the irradiator. A dose of 618 mJ/cm^2^ was administered to each mouse, with an average exposure time of 5 min 42 s. All procedures were performed under sterile conditions in a laminar air-flow unit.

### Lipidomics and metabolomics

Our study utilized the same lipidomics and metabolomics methods developed previously^22^ and as used in various other publications^23–25^. To ensure consistency and improve the understandability of the results presented in this paper, the method description was retained.

### Mouse intra-cutaneous lipid extraction

Lipid extraction was carried out from frozen 3.26–14.7 mg mouse skin biopsies according to a modified version of the original extraction protocol previously published^26^. Methanol (0.75 mL) and methyl-tert-butyl ether (MTBE, 2.5 mL) were added to the samples in 12 mL glass tubes with teflon lined caps. Tissue was homogenized 30 s using the Ultra-Turrax tissue homogenizer (IKA Works Inc., Wilmington, NC, USA). After vortexing for 10 s, the mixture was incubated in an ice-cooled ultrasound bath for 10 min. After addition of further 0.75 mL methanol and 2.5 mL MTBE samples were shaken in an overhead shaker for 10 min at room temperature. After addition of 1.25 mL deionized water and 10 min of additional overhead shaking, the mixture was centrifuged for 10 min at 1350*g* (room temperature) and the upper phase was transferred to a new glass tube. The lower phase was re-extracted with 2 mL of the upper phase of MTBE/methanol/deionized water (10:3:2.5, v/v/v) and again the upper phase was collected, combined with the upper phase from the first extraction^24,25^. Finally, the upper phase was evaporated in a vacuum centrifuge (Thermo Fisher Scientific, Waltham, MA, USA) and dissolved in 500 µL chloroform/methanol (1:1, v/v)^24,25^. For negative measurement 90 µL aliquots were spiked with 86.3 µL internal standards (chloroform/methanol, 1:1,v/v) containing 12:0/13:0 PI, 17:0/20:4 PI, 14:1/17:0 PI, 21:0/22:6 PI, 12:0/13:0 PG, 17:0/20:4 PG, 14:1/17:0 PG, 21:0/22:6 PG (1.5 µM each) and CL-Mix LM 6003 (2.4 µM). The solvent was evaporated under a gentle stream of nitrogen and the sample reconstituted in the same volume of injection solvent isopropanol/chloroform/methanol (90:5:5, v/v/v)^24,25^. For positive measurement 2.1 µL aliquots were spiked with 127.7 µL internal standards (chloroform/methanol, 1:1, v/v) containing PC 12:0/13:0, PC 17:0/20:4, PC 14:1/17:0, PC 21:0/22:6 (1 µM each), PE 12:0/13:0, PE 17:0/20:4, PE 14:1/17:0, PE 21:0/22:6, PS 12:0/13:0, PS 17:0/20:4, PS 14:1/17:0, PS 21:0/22:6 (1.5 µM each),TG-Mix LM 6000 (4 µM each), LPC 17:1 (1 µM), SL-Mix LM6002 (1.5 µM each), CE 19:0 (12 µM), and cholesterol-*d*_7_ (80 µM) and 36 µL of this mix were used for further processing. The solvent was evaporated under a gentle stream of nitrogen and the sample reconstituted in 90 µL injection solvent isopropanol/chloroform/methanol (90:5:5, v/v/v).

### LC–MS/MS lipidomics

Chromatographic separation for sphingolipids was performed as previously described^22^. Briefly, chromatographic separation was performed on a Waters (Waters, Milford, MA, USA) BEH C8 column (100 × 1 mm, 1.7 µm), thermostatted to 50 °C in a Dionex Ultimate 3000 RS ultra-high-pressure liquid chromatography (UHPLC) system. Mobile phase A was deionized water containing 1 vol% of 1 M aqueous ammonium formate (final concentration 10 mmol/L) and 0.1 vol% of formic acid as additives. Mobile phase B was a mixture of acetonitrile/isopropanol 5:2 (v/v) with the same additives. Gradient elution started at 50% mobile phase B, rising to 100% B over 15 min; 100% B were held for 10 min and the column was re-equilibrated with 50% B for 8 min before the next injection. The flow rate was 150 µL/min, the samples were kept at 8 °C and the injection volume was 2 µL^22–25,27^.

The Orbitrap Velos Pro hybrid mass spectrometer (Thermo Fisher Scientific Inc., Waltham, MA, USA) was operated in data dependent acquisition (DDA)^22–25,27,28^. Every sample was acquired once in positive polarity and once in negative using a HESI II ion source. Ion source parameters for positive polarity were as follows: Source Voltage: 4.5 kV; Source Temperature: 275 °C; Sheath Gas: 25 arbitrary units; Aux Gas: 9 arbitrary units; Sweep Gas: 0 arbitrary units; Capillary Temperature: 300 °C^22–25,27,28^. Ion source parameters for negative polarity were as follows: Source Voltage: 3.8 kV; Source Temperature: 325 °C; Sheath Gas: 30 arbitrary units; Aux Gas: 10 arbitrary units; Sweep Gas: 0 arbitrary units; Capillary Temperature: 300 °C. Automatic gain control target value was set to 10^6^ ions to enter the mass analyser, with a maximum ion accumulation time of 500 ms. Full scan profile spectra from *m/z* 320 to 1050 for positive ion mode and from 350 to 1600 in negative ion mode were acquired in the Orbitrap mass analyser at a resolution setting of 100,000 at *m/z* 400^22–25,27,28^. For MS/MS experiments in positive and negative ion mode, the 10 most abundant ions (Top 10) of the full scan spectrum were sequentially fragmented in the ion trap using He as collision gas (CID, Normalized Collision Energy: 50; Isolation Width: 1.5; Activation Q: 0.2; Activation Time: 10) and centroid product spectra at normal scan rate (33 kDa/s) were collected^22–25,27,28^.

### Lipidomics targeted data extraction

LC/MS data were processed using Lipid Data Analyzer (LDA)^27,29^. Briefly, the algorithm identifies lipids with a 3D algorithm, using the three dimensions m/z, retention time, and intensity to correctly integrate peaks, while also considering the isotopic distribution. MS/MS spectra are considered for confirmation of structures by characteristic head group and fatty acyl fragments. Lipids are annotated according to the official international shorthand nomenclature^28^.

### Metabolite standards

The following substances^22–25,27,28^ were used as references for retention times.

Dopamine HCl (56610-5G; ≥ 98.5%), l-ornithine monohydrochloride (75469-25G; ≥ 99.5%), l-prolin (81710-10G; ≥ 99%), taurine (86330-25G; ≥ 99%), creatinine (C4255-10G; ≥ 98%), d-(+)-glucose (G8270-100G; ≥ 99.5%), l-alanine (A7627-1g; ≥ 98%), l-asparagine (A0884-25G; ≥ 98%), l-aspartic acid (A8949-25G; ≥ 99%), l-citrulline (C7629-10MG; ≥ 98%), l-glutamic acid (G1251-100G; ≥ 99%), l-glutamine (G8540-25G; ≥ 99%), l-histidine (H8000-5G; ≥ 99%), l-isoleucine (I2752-1G; ≥ 98%), l-leucine (L8000-25g; ≥ 98%), l-lysine (L5501-1G; ≥ 98%), l-methionine sulfoxide (M1126-1G; NA), l-phenylalanine (P2126-100G; ≥ 98%), l-serine (S4500-1G; ≥ 99%), l-threonine (T8625-1G; ≥ 98%), l-tryptophan (T0254-5G; ≥ 98%), l-tyrosine (T3754-50G; ≥ 98%), l-valine (V0500-1G; ≥ 98%), cholic acid (C1129-25G; ≥ 98%), d-carnitine (544361-1G; ≥ 98%), decanoyl-l-carnitine (50637-10MG; ≥ 94%), Folic acid (F7876-1G; ≥ 97%), hippuric acid (112003-5G; ≥ 98%), l-carnosine (C9625-10MG; ≥ 99%), Palmitoyl-l-carnitine (91503-10MG; ≥ 95%), Riboflavin (R4500-5G; ≥ 98%), valeryl-l-carnitine (04265-10MG; ≥ 95%), adenine (A8626-1G; ≥ 99%), cytidine (C122106-1G; ≥ 99%), cytosine (C3506-1G; ≥ 99%), d-arginine (A2646-250MG; ≥ 98%), choline (C7017-10MG, ≥ 99%), a-tocopherol (T3251-5G, ≥ 96%), adenosine (A9251-1G, ≥ 99%), and methionine (M9625-5G, ≥ 98%) standards were purchased from Sigma-Aldrich (St. Louis, MO, USA), and Estradiol (E0950-000; NA) from Steraloids (Newport, RI, USA).

### Mouse intra-cutaneous metabolite extraction

Sample preparation was performed according to Bruce et al*.*^30^, with some modifications. Briefly, mouse skin was homogenized 30 s on ice (4 °C) in 375 µL MeOH/H_2_O (1/1, v/v) using the Ultra-Turrax tissue homogenizer (IKA Works Inc., Wilmington, NC, USA). Proteins were precipitated by adding a 3:1 volume of ice-cold acetonitrile/methanol/acetone (1/1/1, v/v/v) and using the Ultra-Turrax again for 30 s on ice. After an additional precipitation step at 4 °C for 60 min, samples were centrifuged at 1 419 relative centrifugal force (rcf) for 10 min (Hereaus Biofuge pico, Hanau, Germany). The resultant supernatants were aspirated into clean Eppendorf tubes and evaporated under a gentle stream of nitrogen gas at room temperature. Dry extracts were re-suspended in acetonitrile/water (1/1, v/v) to 200 µL sample volume, respectively, and immediately stored at − 80 °C until further analysis. To evaluate data processing and retention times a mixture of metabolite standard was measured in addition to biological samples. The final injection concentration of these standards was 10 µM solved in acetonitrile/water (1:1, v/v). and the sample reconstituted in 250 µL injection solvent isopropanol.

### LC–MS/MS metabolomics

For all samples a full-scan mass-spectrometric interrogation of each sample’s small molecule was achieved by Dionex Ultimate 3000 UHPLC (ultra-high performance liquid chromatography)-Orbitrap Velos Pro hybrid mass spectrometer (Thermo Fisher Scientific, Waltham, MA, USA). Separation was performed on an Acquity UPLC BEH Amide column (2.1 mm × 150 mm, 1.7 µm) (Waters Corporation, Milford, USA), thermostated to 40 °C. Mobile phase A was 97% ACN + 3% H_2_O + 0.1 mM NH_4_COOH + 0.16% HCOOH. Mobile phase B was H_2_O + 0.1 mM NH_4_COOH + 0.16% HCOOH, and autosampler wash solution ACN/H_2_O (1:1, v/v). The starting point of gradient elution was 5% mobile phase B and increased up to 30% over 30 min. Mobile phase B was reset to start conditions over a minute and re-equilibrated for 9 min before 2 µL of the next sample was re-injected. Flow rate was 200 µL min^−1^ and samples were thermostatted at 8 °C in the autosampler. The Orbitrap Velos Pro operated in data dependent acquisition mode using a HESI II ion source. Full scan spectra from *m/z* 60 to 1600 were acquired in the Orbitrap with a resolution of 100 000 (*m/z* 400) in positive mode and the 10 most abundant ions of the full-scan spectrum were sequentially fragmented with CID (normalized collision energy, 50) and analyzed in the linear ion trap. Isolation width was 1.5, activation Q: 0.2; activation time: 10, and the centroided product spectra at normal scan rate were collected. The exclusion time was set to 11 s and as lock mass a polysiloxane with *m/z* 536.16537 was chosen. The following source parameters were used: Source voltage: 4.5 kV, source temperature: 275 °C, sheath gas: 25 arbitrary units, aux gas: eight arbitrary units, sweep gas: zero arbitrary units, capillary temperature: 300 °C^22–25,27,28^.

### Lipidomics and metabolomics untargeted data extraction

Raw MS data were processed on MS-DIAL software v4.47^31^. First, MS data (.raw) were converted to mzML format with msConvert tool (http://proteowizard.sourceforge.net). After conversion, MS-DIAL software was used for feature detection, deconvolution of spectra, peak alignment, and lipid identification^32^. Briefly, ionization mode (positive and negative), data type (centroided) was selected. For peak detection process, the peak intensity threshold was set up at 1000 of amplitude and the mass slice was set up at 0.1 Da. Linear weighted moving average method was preferred for smoothing (smoothing level 3 scan) and minimum peak width was adjusted to 5. For deconvolution parameters, sigma window value was set up at 0.5. For alignment parameters setting, retention time and MS^1^ mass tolerance was selected at 0.05 min and 0.015 Da, respectively. Gap filling function was used for missing value interpolation. Adduct ion setting including protonated molecule, ammonium adduct ion and deprotonated molecule was considered for positive and negative ion mode respectively. For lipid species annotation, MS finder vs 3.52 was used^33^.

### Lipidomics and metabolomics statistical analysis

Data visualisation and statistical analysis were performed with R v4.2 (using the packages dplyr, openxlsx, readxl, stringr, tibble, tidyr, ggplot2, ggpubr, ggpmisc, ggrepel, ggforce, colorspace, grid, scales, missMDA, mixOmics, nlme, emmeans, rstatix) and TIBCO Spotfire v12.0.1, TIBCO, Palo Alto, CA. All 111 targeted lipids were normalized to their corresponding internal standards and tissue weight of the skin sample yielding absolute quantitative values in pmol/mg. All 34 targeted metabolites peak areas were integrated yielding arbitrary units and were normalized to tissue weight of the skin sample. Untargeted lipids and metabolites were extracted in arbitrary units. Both targeted and untargeted data did not correlate with tissue weight as checked by Pearson correlation.

Untargeted metabolites and lipids data were trimmed with the very conservative and outlier robust metric MAD score^34^ of > 5 removing very severe single value outliers (< 1% data). All features were further filtered for data quality, excluding 126 features with no median standard deviation (MSD) and 146 features with > 20% of all values with very low intensity (< 10 a.u.) retaining 3663 features for further analysis. Targeted and untargeted data were combined to a total of 3808 metabolites and lipids. The dataset contained no zeros and < 2% missing values. All data were log_10_-transformed (LOG) for statistical analysis to improve distribution and scedasticity. Distribution and scedasticity were investigated with Kolmogorov–Smirnov test and Brown–Forsythe Levene-type test, respectively. All included LOG metabolites and lipids were sufficiently normal and > 95% were sufficiently homoscedastic.

Only statistical methods were applied that were able to provide unbiased estimates for the here unbalanced group sizes pooled from independent experiments. Partial least-squares discriminant analysis (PLS-DA) with all features (LOG transformed) was performed after imputation with *missMDA::imputePCA()* using 6 components. Data was further centred and scaled to unit variance with *mixOmics::plsda()* and the multilevel option was used to correct for laboratory differences (factor Exp_ID) for control mice. For readability, the axes of PLS-DA scores and loadings plots have been multiplied by − 1, as indicated in the figures, because the classification results or interpretation of the data are independent of direction. Significances of group differences were evaluated with *mixOmics::auroc()*.

Linear mixed models (LME) were fitted with *nlme::lme()* with maximum likelihood (ML) on LOG data (LOGLME) for the factor *group* (control UV−, control UV+, GF UV−, GF UV+, Disinfected UV−, Disinfected UV+) and the laboratory as random factor (~ 1|Exp_ID), analog to PLS-DA multilevel option. This renders the approach nonlinear mixed models, however due to the name similarity to the *nlme* package name we used LOGLME for clarity. Criteria for model performance and suitability were lower AIC (Akaike information criterion; relative estimate of information loss), higher log-likelihood (goodness of fit), significance in log likelihood ratio test comparing two models, quality of Q–Q plots and randomness in residual plots. The addition of the random factor had no influence on model performance or results in ~ 84% of all metabolites and lipids but improved performance significantly for the remainder 16%. For consistency and comparability between metabolites and lipids only results from models with random factor are reported. Post-hoc pairwise comparisons were readout with *emmeans::emmeans*, and p ≤ 0.05 were considered statistically significant. All reported p-values were adjusted for multiple testing according to Benjamini–Hochberg (BH) (R function *stats::p.adjust*).

Pathway analysis was performed online with the functional analysis module from MetaboAnalyst 5.0^35^ using mummichog-based annotation for all positive, untargeted features providing exact masses and retention times with a mass tolerance of 5 ppm without further feature filtering. Data was LOG transformed but no further normalizations or scalings were performed in Metaboanalyst. Normalized enrichment score (NES) analysis was performed for each pairwise group comparison based on the 1388–1391 compound matches with the GSEA algorithm on the mummichog original MFN library for pathways with at least 3 entries.

## Supplementary Information

Supplementary Table 1.

Supplementary Figures.

## Data Availability

Untargeted lipidomics and metabolomics data have been deposited to the EMBL-EBI MetaboLights database with the identifier MTBLS6969. The data sets can be accessed here: https://www.ebi.ac.uk/metabolights/MTBLS6969.

## References

[1] Bernard JJ, Gallo RL, Krutmann J (2019). Photoimmunology: How ultraviolet radiation affects the immune system. Nat. Rev. Immunol..

[2] Vieyra-Garcia PA, Wolf P (2021). A deep dive into UV-based phototherapy: Mechanisms of action and emerging molecular targets in inflammation and cancer. Pharmacol. Ther..

[3] Elpa DP, Chiu HY, Wu SP, Urban PL (2021). Skin metabolomics. Trends Endocrinol. Metab..

[4] Cibrian D, de la Fuente H, Sanchez-Madrid F (2020). Metabolic pathways that control skin homeostasis and inflammation. Trends Mol. Med..

[5] Chen H (2022). Skin microbiome, metabolome and skin phenome, from the perspectives of skin as an ecosystem. Phenomics.

[6] Patra V, Wagner K, Arulampalam V, Wolf P (2019). Skin microbiome modulates the effect of ultraviolet radiation on cellular response and immune function. iScience.

[7] Flowers L, Grice EA (2020). The skin microbiota: Balancing risk and reward. Cell Host Microbe.

[8] Hawkshaw NJ (2020). UV radiation recruits CD4(+)GATA3(+) and CD8(+)GATA3(+) T cells while altering the lipid microenvironment following inflammatory resolution in human skin in vivo. Clin. Transl. Immunol..

[9] Tse BCY, Byrne SN (2020). Lipids in ultraviolet radiation-induced immune modulation. Photochem. Photobiol. Sci..

[10] Byrd AL, Belkaid Y, Segre JA (2018). The human skin microbiome. Nat. Rev. Microbiol..

[11] Chu X (2021). Integration of metabolomics, genomics, and immune phenotypes reveals the causal roles of metabolites in disease. Genome Biol..

[12] Boo YC (2022). Ascorbic acid (vitamin C) as a cosmeceutical to increase dermal collagen for skin antiaging purposes: Emerging combination therapies. Antioxidants (Basel).

[13] Gisondi P, Fantuzzi F, Malerba M, Girolomoni G (2007). Folic acid in general medicine and dermatology. J. Dermatolog. Treat..

[14] Maceyka M, Spiegel S (2014). Sphingolipid metabolites in inflammatory disease. Nature.

[15] Yu G, Xu C, Zhang D, Ju F, Ni Y (2022). MetOrigin: Discriminating the origins of microbial metabolites for integrative analysis of the gut microbiome and metabolome. iMeta.

[16] Helferich WG, Denison MS (1991). Ultraviolet photoproducts of tryptophan can act as dioxin agonists. Mol. Pharmacol..

[17] Randhawa M, Sangar V, Tucker-Samaras S, Southall M (2014). Metabolic signature of sun exposed skin suggests catabolic pathway overweighs anabolic pathway. PLoS One.

[18] Kremslehner C (2020). Imaging of metabolic activity adaptations to UV stress, drugs and differentiation at cellular resolution in skin and skin equivalents—Implications for oxidative UV damage. Redox Biol..

[19] Buchberger AR, DeLaney K, Johnson J, Li L (2018). Mass spectrometry imaging: A review of emerging advancements and future insights. Anal. Chem..

[20] Meintani DG, Chatzimitakos TG, Kasouni AI, Stalikas CD (2022). Untargeted metabolomics of human keratinocytes reveals the impact of exposure to 2,6-dichloro-1,4-benzoquinone and 2,6-dichloro-3-hydroxy-1,4-benzoquinone as emerging disinfection by-products. Metabolomics.

[21] Roux PF, Oddos T, Stamatas G (2022). Deciphering the role of skin surface microbiome in skin health: An integrative multiomics approach reveals three distinct metabolite-microbe clusters. J. Investig. Dermatol..

[22] Triebl A, Trotzmuller M, Hartler J, Stojakovic T, Kofeler HC (2017). Lipidomics by ultrahigh performance liquid chromatography-high resolution mass spectrometry and its application to complex biological samples. J. Chromatogr. B Analyt. Technol. Biomed. Life Sci..

[23] Zullig T (2020). A metabolomics workflow for analyzing complex biological samples using a combined method of untargeted and target-list based approaches. Metabolites.

[24] Zandl-Lang M (2022). Changes in the cerebrospinal fluid and plasma lipidome in patients with Rett syndrome. Metabolites.

[25] Zhang Y (2021). Asymmetric opening of the homopentameric 5-HT(3A) serotonin receptor in lipid bilayers. Nat. Commun..

[26] Matyash V, Liebisch G, Kurzchalia TV, Shevchenko A, Schwudke D (2008). Lipid extraction by methyl-tert-butyl ether for high-throughput lipidomics. J. Lipid Res..

[27] Hartler J (2011). Lipid Data Analyzer: Unattended identification and quantitation of lipids in LC-MS data. Bioinformatics.

[28] Liebisch G (2013). Shorthand notation for lipid structures derived from mass spectrometry. J. Lipid Res..

[29] Hartler J (2017). Deciphering lipid structures based on platform-independent decision rules. Nat. Methods.

[30] Bruce SJ (2009). Investigation of human blood plasma sample preparation for performing metabolomics using ultrahigh performance liquid chromatography/mass spectrometry. Anal. Chem..

[31] Tsugawa H (2020). A lipidome atlas in MS-DIAL 4. Nat. Biotechnol..

[32] Tsugawa H (2019). A cheminformatics approach to characterize metabolomes in stable-isotope-labeled organisms. Nat. Methods.

[33] Lai Z (2018). Identifying metabolites by integrating metabolome databases with mass spectrometry cheminformatics. Nat. Methods.

[34] Leys C, Ley C, Klein O, Bernard P, Licata L (2013). Detecting outliers: Do not use standard deviation around the mean, use absolute deviation around the median. J. Exp. Soc. Psychol..

[35] Pang Z (2021). MetaboAnalyst 5.0: Narrowing the gap between raw spectra and functional insights. Nucleic Acids Res..

