# Supplementary material for: Ultraviolet exposure regulates skin metabolome based on the microbiome
Source: Sci Rep. 2023 May 3;13:7207. doi: 10.1038/s41598-023-34073-3 (PMC10156686; doi:10.1038/s41598-023-34073-3)
Supplement: Supplementary file 2 — Supplementary Figures. [file 41598_2023_34073_MOESM2_ESM.docx]

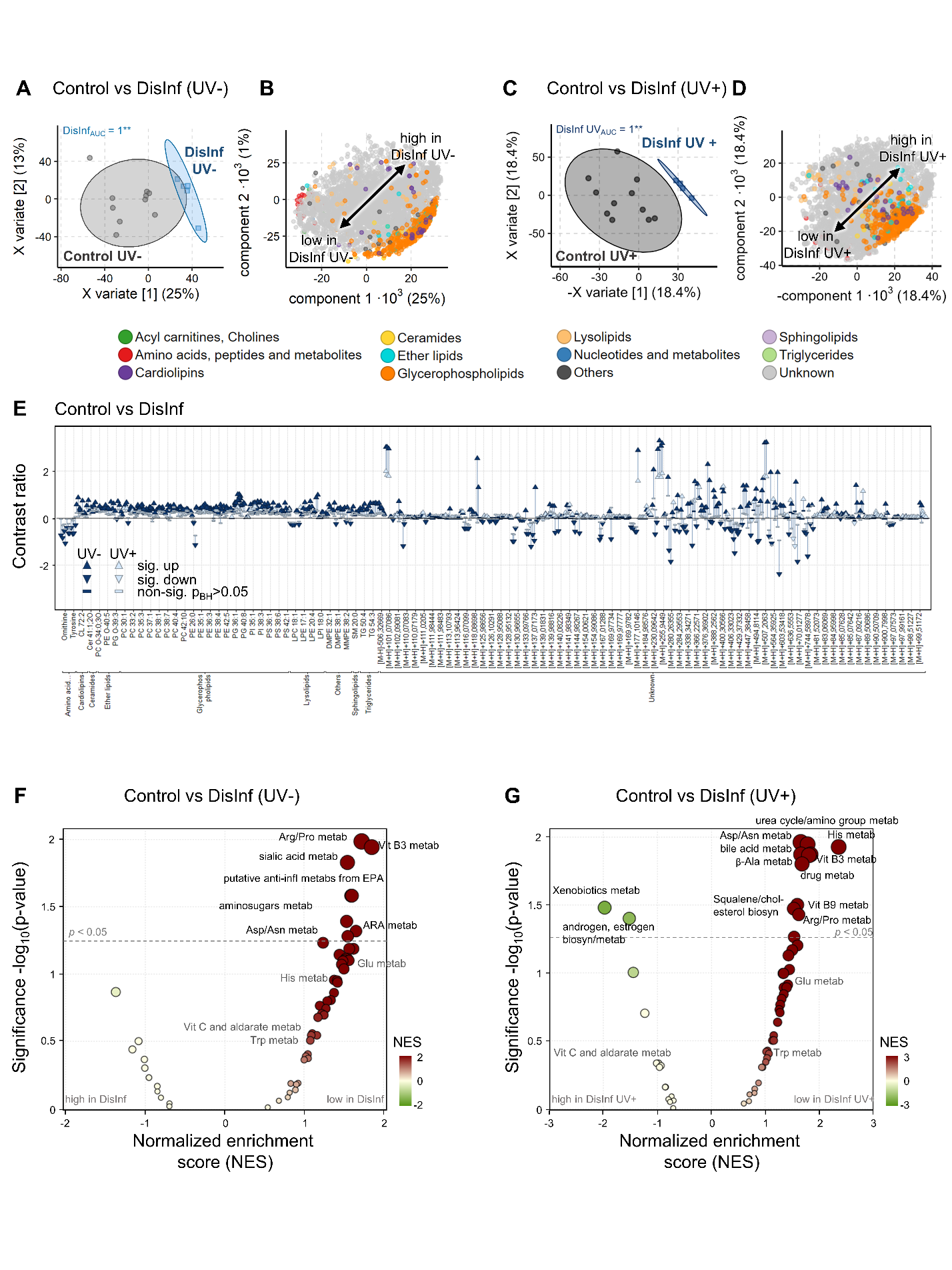
**Figure S1. Cutaneous metabolome strongly depends on the presence of local microbiome before and after UVB exposure.**

1. PLS-DA scores plot investigating the difference of cutaneous metabolome in the absence of local microbiome (disinfected) before UVB exposure compared to control mice. Points and ellipse as in Fig. 1. ROC analysis with X variate 1-3 found the metabolomes to differ significantly (p < 0.01) with an AUC of 1.
2. Corresponding PLS-DA loadings plot to A) (points = metabolites as in Fig. 1) showing that unknown metabolites strongly differ after UV exposure.
3. PLS-DA scores plot investigating the difference of cutaneous metabolome in the absence of local microbiome (disinfected) after UVB exposure compared to control mice. Points and ellipse as in Fig. 1. ROC analysis with X variate 1-3 found the metabolomes to differ significantly (p < 0.01) with an AUC of 1.
4. Corresponding PLS-DA loadings plot to A) (points = metabolites as in Fig. 1) showing that unknown metabolites strongly differ after UV exposure.
5. Dumbbell plot of all significant LOGLME metabolites (p < 0.05) in any of the two comparisons: disinfected mice vs control mice without UV exposure (dark blue) or with UV exposure (light blue). The plot shows the strength of metabolic changes along the y-axis, significance is encoded in shapes (p < 0.05) indicating significant increases, decreases or non-significances. Note how there are much fewer significant differences after UV exposure (light blue).
6. Functional analysis of unknown metabolites in the absence of local microbiome (disinfected) before UVB exposure compared to control mice. Only significantly impacted pathways are labelled (p<0.05).
7. Functional analysis of unknown metabolites in the absence of local microbiome (disinfected) after UVB exposure compared to control mice. Significantly impacted pathways are labelled above the dotted line (p<0.05). N = 5-10 mice per experimental group. Data pooled from two independent experiments.


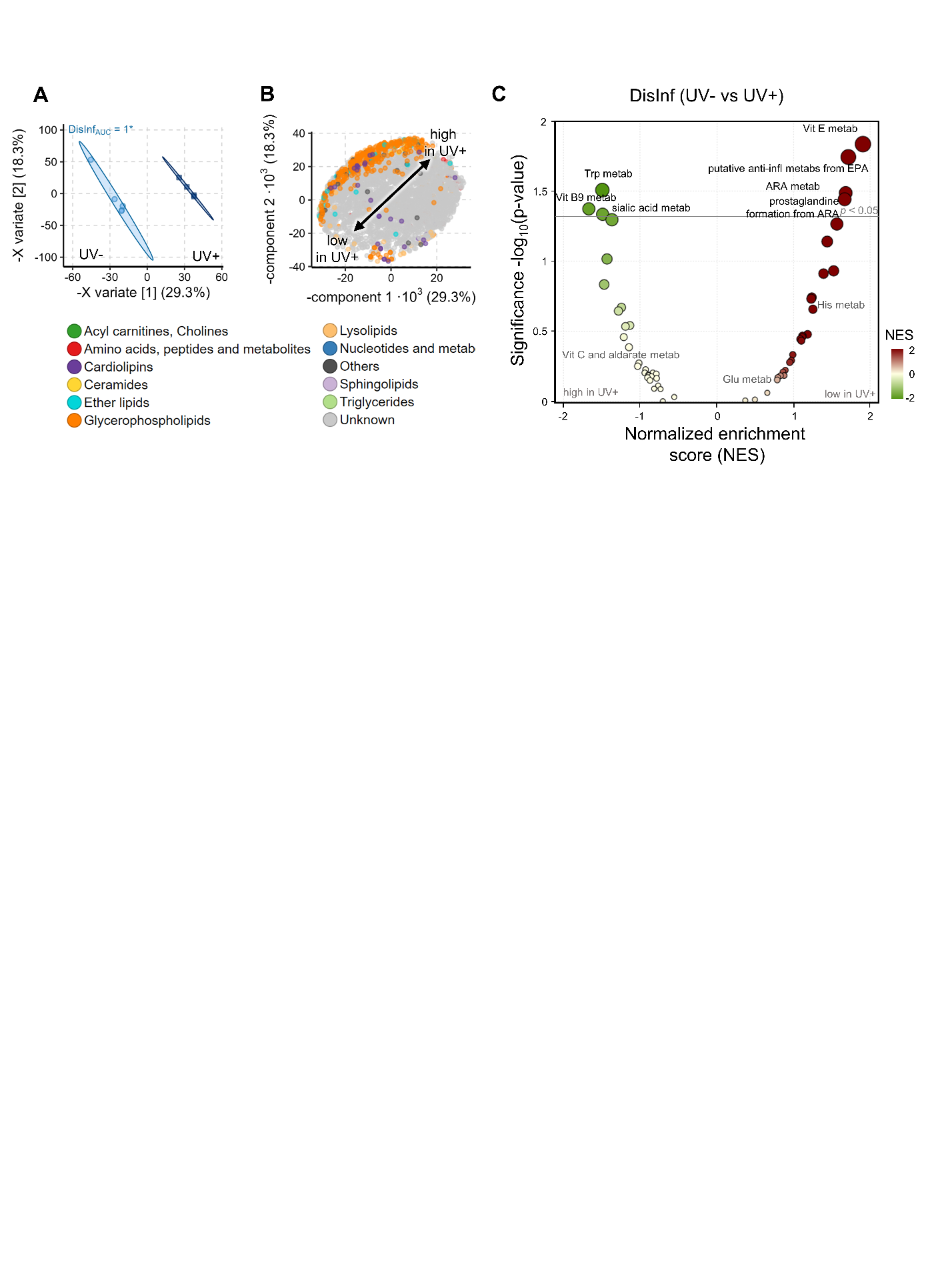


### Figure S2. UVB exposure reduces intra-cutaneous metabolic differences induced by presence/absence of local microbiome.

1. PLS-DA scores plot investigating the difference of cutaneous metabolome induced by UVB exposure in disinfected mice. Points and ellipse as in Fig. 1. ROC analysis with X variate 1-3 found the metabolomes to differ significantly (p < 0.01) with an AUC of 1.
2. Corresponding PLS-DA loadings plot to A) (points = metabolites as in Fig. 1) showing that unknown metabolites strongly differ after UV exposure.
3. Functional analysis of unknown metabolite changes induced by UVB exposure in disinfected mice. Significantly impacted pathways are labelled above the dotted line (p<0.05). N = 5-10 mice per experimental group. Data pooled from two independent experiments.
